# MITOCHONDRIA: POWERHOUSE, SLAUGHTERHOUSE, AND SPEAKER OF THE HOUSE

**DOI:** 10.1093/geroni/igac059.1142

**Published:** 2022-12-20

**Authors:** Changhan Lee

**Affiliations:** University of Southern California, Los Angeles, California, United States

## Abstract

The nuclear and mitochondrial genomes have co-evolved since the union forged 1-2 billion years ago between our ancestral cell and free-living bacteria. The bi-genomic system is coordinated by close communication between the two genome-possessing organelles. More recently, peptides that are encoded in the mitochondrial genome have been identified and shown to communicate mitochondrial messages. In this symposium, I will discuss MOTS-c as a mitochondrial-encoded communication factor in the context of aging. MOTS-c can translocate to the nucleus under metabolic stress to directly regulate adaptive nuclear gene expression. In humans, MOTS-c levels increase in skeletal muscle and in circulation upon exercise. In mice, MOTS-c treatment significantly reversed physical decline in aged mice (22 mo.), including doubling in treadmill running time. Studies from our lab and others point to a novel class of mitochondrial-encoded longevity genes that coordinate cellular homeostasis.

